# Predictors of Failed Conscious Sedation in Patients Undergoing an Outpatient Colonoscopy and Implications for the Adenoma Detection Rate

**DOI:** 10.1038/s41598-020-59189-8

**Published:** 2020-02-07

**Authors:** Benjamin E. Cassell, Kristina Ross, Tae Y. Chang, Gregory L. Austin

**Affiliations:** 1 The Division of Gastroenterology and Hepatology, The University of Colorado School of Medicine, Aurora, CO USA; 2 Department of Internal Medicine, The University of Arizona College of Medicine – Phoenix, Phoenix, AZ USA

**Keywords:** Colonoscopy, Gastroenterology, Outcomes research

## Abstract

Guidelines to triage patients to conscious sedation (CS) or monitored anaesthesia care (MAC) for colonoscopy do not exist. We aimed to identify the CS failure rate, predictors of failure, and its impact on the adenoma detection rate (ADR). Strict (based on patient experience) and expanded (based on doses of sedative medications) definitions of CS failure were used. Patient and procedure-related variables were extracted. Multivariable logistic regression identified predictors for CS failure and the ADR. Among 766 patients, 29 (3.8%) and 175 (22.8%) patients failed CS by strict and expanded definitions, respectively. Female gender (OR 3.50; 95% CI: 1.37–8.94) and fellow involvement (OR 4.15; 95% CI: 1.79–9.58) were associated with failed CS by the strict definition. Younger age (OR 1.27, 95% CI: 1.07–1.49), outpatient opiate use (OR 1.71; 95% CI 1.03–2.84), use of an adjunct medication (OR 3.34; 95% CI: 1.94–5.73), and fellow involvement (OR 2.20; 95% CI: 1.31–3.71) were associated with failed CS by the expanded definition. Patients meeting strict failure criteria had a lower ADR (OR 0.30; 95% CI: 0.12–0.77). Several clinical factors may be useful for triaging to MAC. The ADR is lower in patients meeting strict criteria for failed CS.

## Introduction

Procedural sedation for gastrointestinal endoscopy is a near universal practice in the United States^1^. This is typically accomplished in one of two ways: standard conscious sedation (CS), in which intravenous sedation is administered by a nurse under direct orders from the endoscopist, or monitored anaesthesia care (MAC) where sedation is administered by an anaesthesiology professional, usually in the form of intravenous propofol^1,2^. MAC offers many potential benefits including improved patient satisfaction and quicker sedation and recovery times^3^. However, MAC has been associated with an increased risk of complications during endoscopy when compared to CS and has been shown to increase the cost of endoscopy by approximately 20%^4–7^.

For these reasons, many institutions reserve MAC for patients undergoing routine endoscopic procedures who are felt to be unlikely to tolerate CS either due to severe or unstable medical comorbidities or predicted high tolerance to the typical sedation regimen. However, the decision about which patients should be triaged to MAC or CS is currently not based on any standardized, objective criteria. While excellent guidelines from professional gastroenterology and anaesthesiology societies make some recommendations based on certain high risk factors, they do not make explicit, concrete recommendations to help triage patients to sedation type^3,8,9^. Furthermore, while some scoring systems exist they have not been widely adopted^10,11^. Additionally, the rate at which patients who are triaged to CS and do poorly or fail is unknown.

While failure of CS certainly will lead to a worse patient experience and increased costs if the procedure cannot be completed and has to be rescheduled, its impact on colonoscopy quality is not clear. The adenoma detection rate (ADR) has been described as “the single most important quality measure in colonoscopy”^12^. Given its importance, there have been countless studies analysing predictors of ADR, but to our knowledge the link between inadequate procedural sedation and ADR has not been previously examined.

For this study, we sought the answer to these questions by looking at colonoscopies performed at a hospital-based academic outpatient endoscopy unit. We aimed to identify the rate of failure of CS, as well as patient and procedural factors that were associated with failed CS. Finally, we wanted to determine the impact failed CS had on ADR.

## Methods

Consecutive adult outpatients presenting for colonoscopy at an academic hospital-based endoscopy unit between July 1, 2015 and November 12, 2015 were included in the study. Patients who had unsedated procedures, had an EGD in addition to their colonoscopy in the same endoscopy session, or who had missing study variables were excluded. All other outpatient colonoscopies performed by endoscopists who performed at least 25 colonoscopies during the study period were included. The following patient variables were obtained from the electronic medical record (Epic, Verona, WI): age, gender, ethnicity, body mass index (BMI), American Society of Anesthesiologists (ASA) Classification, outpatient use of opiate medications, outpatient use of benzodiazepines, use of psychoactive medications, history of cirrhosis, pelvic surgery, Roux-en-Y gastric bypass, obstructive sleep apnea (OSA) and use of continuous positive airway pressure (CPAP), alcohol abuse (defined as greater than 14 drinks per week for men and greater than 7 drinks per week for women)^13^, illicit substance use and history of prior problems with sedation. The following procedure-related variables were extracted from the endoscopy reporting software (Provation, Minneapolis, MN): procedure indication, the individual endoscopist (n = 8), furthest extent of colonoscope advancement, medication dosages, use of adjunctive medications such as diphenhydramine and promethazine, difficulty of procedure, and trainee involvement in procedure.

We used two definitions for failure of CS. There was a “strict” definition which included only those patients in which the field in the procedure report indicated that the patient tolerated the procedure less than “well”. This included “tolerated procedure fairly well”, “tolerated procedure”, and “tolerated procedure poorly”. For this strict definition of failed CS, we also included patients who received a recommendation in the procedure report that anaesthesia should be used for future procedures. Our “expanded” definition for failed CS included all patients meeting the “strict” criteria as well as patients who required more than 5 mg of midazolam and/or more than 200 mcg of fentanyl. Colonoscopies that failed to reach the cecum were included. Of the 29 who met the strict definition for failed CS, the cecum was not reached in two patients with only one occurring because of inadequate sedation. Failure to reach the cecum was not a criterion for failed conscious sedation.

Information regarding the adenoma detection rate (ADR) is collected routinely for all colonoscopies at the University of Colorado Hospital. In addition to recording the most histologically advanced polyp, the total number of polyps removed, and the size of largest polyp are also recorded. The information from this dataset was linked to the procedures in our cohort so that polyp data could be linked to sedation parameters and compared between subjects who did and did not fail CS. For the purposes of this study, ADR was defined as the percentage of patients with at least one tubular adenoma or sessile serrated adenoma on their colonoscopy regardless of indication. This study was approved by the Colorado Multiple Institutional Review Board.

### Statistical analysis

All data were entered into and analysed using STATA 10.0 statistical software (StataCorp, College Station, Texas). Univariate analyses of categorical variables were performed using the chi-square test and continuous variables were analysed using Student’s T-test. Multivariable logistic regression analysis was used to identify significant predictors for both definitions of failed CS. The final model regression model for failed CS using the strict definition included gender, use of an adjunct medication for sedation (e.g., diphenhydramine), and fellow involvement in the colonoscopy. The final model regression model for failed CS using the expanded definition included age, gender, use of an adjunct sedation medication, procedure indication, outpatient opiate use, outpatient benzodiazepine use, fellow involvement, and the individual endoscopist. Ethnicity, BMI, ASA classification, psychoactive medication use, history of cirrhosis, prior pelvic surgery, prior roux-en-y gastric bypass, OSA with or without CPAP use and alcohol or illicit substance use/abuse were excluded from both multivariable models as these were found to be non-significant (p > 0.1) in univariate analysis. Separate predictive scores for each definition for failure of CS and corresponding receiver operating curves (ROC) were generated. The predictive score for the strict definition of CS was calculated using the regression coefficients from the multivariable model including the variables gender, use of an adjunct medication, and fellow involvement. The predictive score for the expanded definition of CS was calculated using the regression coefficients from the multivariable model that included age, use of an adjunct medication, outpatient opiate use, outpatient benzodiazepine use, and fellow involvement. Although difficulty of procedure was significant in univariate analysis for both definitions this was left out of the predictive scores as this is not something that can be known prior to the procedure.

We examined two multivariable regression models for the ADR; one model incorporated failure of CS using the strict definition and the other model incorporated failure of CS using the expanded definition. The two models were otherwise identical and included age, body mass index, gender, procedure indication (diagnostic versus screening/surveillance), fellow involvement, and the individual endoscopist. The same covariates included in the ADR models were utilized in linear regression models to assess differences in the size of the largest polyp and the total number of polyps as they relate to failure of conscious sedation.

## Results

### Failure of conscious sedation

A total of 766 patients underwent colonoscopy during our study period and met inclusion criteria for analysis. Using the strict definition, 29 patients (3.8%) failed CS. Of these 7 had a specific recommendation to perform future procedures with MAC. The overall cecal intubation rate was 98.9%, the rates for patients who failed (by any definition) or did not fail were 98.3% and 99.1% respectively. Only 1 patient in which a recommendation for MAC was made had a procedure where the scope was not advanced to the cecum.

In univariate analysis (Table 1), female gender (p = 0.001), use of adjunct sedation medications (p < 0.001), and fellow involvement (p = 0.003) were associated with a higher likelihood of failed CS using the strict criteria. Using the expanded definition, 175 patients (22.8%) failed CS. Univariate analysis demonstrated that younger age (p < 0.001), female gender (p = 0.024), use of adjunct sedation medications (p < 0.001), a diagnostic indication for the colonoscopy (p < 0.001), outpatient opiate use (p0.006), outpatient benzodiazepine use (p = 0.002), and fellow involvement (p = 0.001) were associated with failed CS. Any procedural difficulty encountered by the endoscopist was also associated with failure of CS by both definitions (p < 0.001 for both). To further explore the effect of fellow involvement, we evaluated whether year of training was associated with either the strict or expanded definition of failed conscious sedation. The rates of failed conscious sedation were similar across the three years of fellowship for both the strict definition (p = 0.178) and expanded definition (p = 0.308).

**Table 1:** Patient Demographics for Strict and Expanded Criteria for Failed Conscious Sedation.

	Strict Definition			Expanded Definition		
	Did Not Fail (N = 737)	Failed (N = 29)	p-value	Did Not Fail (N = 591)	Failed (N = 175)	p-value
Age (Mean ± S.D.), y	58.3 ± 11.5	57.9 ± 16.9	0.842	59.6 ± 10.7	54.0 ± 13.9	<0.001
Gender (% Female)	48.4	79.3	0.001	47.4	57.1	0.024
Procedure Not Difficult (%)^†^	7.2	44.8	<0.001	4.9	21.1	<0.001
Use of Adjunct Medications for Sedation (%)	15.7	44.8	<0.001	11.5	34.9	<0.001
Screening/Surveillance Indication (%)	77.5	69.0	0.278	80.4	66.3	<0.001
Outpatient Opiate Use (%)	12.9	24.1	0.082	11.5	19.5	0.006
Outpatient Benzodiazepine Use (%)	9.3	13.8	0.412	7.6	15.5	0.002
Body Mass Index (Mean ± S.D.), kg/m^2^	27.8 ± 5.3	27.2 ± 6.8	0.559	27.7 ± 5.0	28.0 ± 6.4	0.542
Fellow Involvement (%)	14.2	34.5	0.003	12.7	22.9	0.001
Use of Psychotropic Medications (%)	23.3	27.6	0.596	22.2	27.7	0.133
History of Illicit Substance Use (%)	6.3	6.9	0.888	5.9	7.4	0.473
History of Alcohol Abuse (%)	5.7	3.4	0.605	6.3	3.4	0.152
ASA Classification 3	7.2	3.4	0.547	6.9	7.4	0.251

†As rated by endoscopist on a scale of: “Performed with ease”, “Not difficult”, “Somewhat difficult”, “Technically difficult”, “Difficult procedure”.

**Figure 1:**
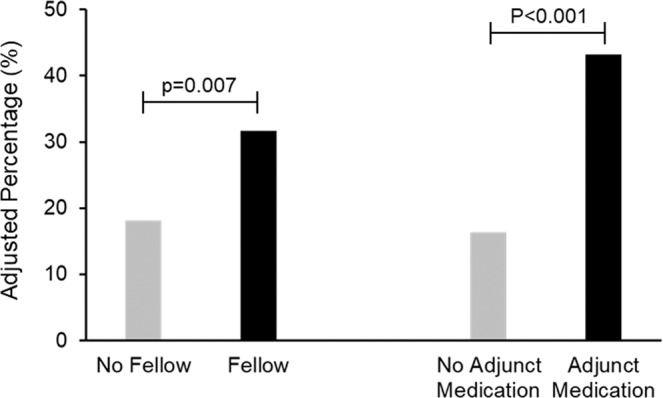
Adjusted Percentage of Patients who met the Expanded Definition for Failed Conscious Sedation by Inolvement of a Fellow^†^ and by Use of Adjunct Medication^‡^ for Sedation. ^†^Fellow involvement adjusted for age, gender, indication, body mass index, use of an adjunct medication and individual endoscopy. ^‡^Adjunct medication use adjusted for age, gender, indication, fellow involvement and individual endoscopist.

**Figure 2:**
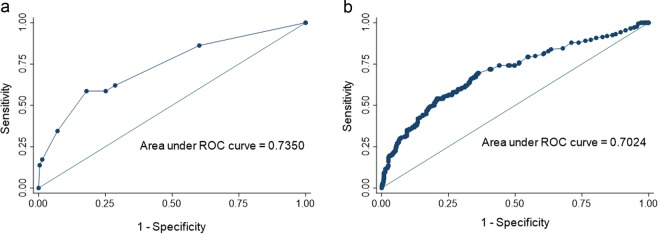
Receiver Operating Curves for Predictive Model for Strict (2a)^†^ and Expanded (2b)^‡^ Definition of Failure of Conscious Sedation. ^†^ROC for strict definition calculated using regression coefficients for gender, use of an adjunct medication, and fellow involvement. ^‡^ROC for expanded definition calculated using regression coefficients for age, use of an adjunct medication, outpatient opiate use, outpatient benzodiazepine use, and fellow involvement.

In multivariable logistic regression analysis (Table 2), female gender (OR 3.50; 95% CI: 1.37–8.94) and fellow involvement (OR 4.15; 95% CI: 1.79–9.58) were associated with failed CS by the strict definition. Interestingly, use of an adjunct medication for sedation was associated with increased odds of failing CS by the strict definition (OR 3.91; 95% CI: 1.76–8.65) independent of outpatient opiate or benzodiazepine use. Outpatient opiate (p = 0.14) and benzodiazepine (p = 0.98) use were not associated with increases odds of failing CS by the strict definition. For the expanded definition (Fig. 1), younger age (OR 1.27 per 10-year decrease, 95% CI: 1.07–1.49), outpatient opiate use (OR 1.71; 95% CI 1.03–2.84), and fellow involvement (OR 2.20; 95% CI: 1.31–3.71) were associated with increased odds of failed CS. Interestingly, use of an adjunct sedation medication was also associated with increased odds of failed conscious sedation (OR 3.34; 95% CI: 1.94–5.73). Outpatient benzodiazepine use was associated with a non-significant increase in the odds of failing CS by the strict definition (OR 1.72; 95% CI: 0.98–3.01). In the multivariable model, gender (p = 0.32) and having a diagnostic indication (p = 0.28) were not associated with increased odds of failed CS. As mentioned previously, difficulty of procedure was not included in the final multivariable models given inability to know procedure difficulty prior to the start of the procedure. However, inclusion of procedure difficulty in the multivariable models would not have substantially altered the strong associations of adjunct medication use and fellow involvement. Risk scores for the strict definition and expanded definition were calculated and receiver operating curves for the strict and expanded definition outcomes were generated (Fig. 2). These models had an area under the ROC curve of 0.735 and 0.702 for the strict and expanded definitions, respectively.

**Table 2:** Multivariable Logistic Regression for Predictors of Failed Conscious Sedation by Strict and Expanded Definitions.

	Strict Definition			Expanded Definition*		
	OR	95%CI	p-value	OR	95%CI	p-value
Male Gender	0.24	0.10–0.61	0.002	0.83	0.57–1.20	0.57
Outpatient Opiate Use	2.42	0.97–6.02	0.058	1.71	1.03–2.84	0.038
Fellow Involvement in Colonoscopy	3.78	1.66–8.61	0.002	2.2	1.31–3.71	0.003
Age (per 10 years)				0.98	0.96–0.99	0.005
Adjunct Med Used				3.34	1.94–5.73	<0.001
Diagnostic Indication				1.28	0.82–2.01	0.276
Outpatient Benzo Use				1.72	0.98–3.01	0.057

*Expanded definition was also controlled for each of the 8 individual faculty endoscopists, none of whom were significant predictors of failed CS by either definition.

### Impact of failed conscious sedation on the adenoma detection rate

Subjects meeting the strict definition for failure of conscious sedation had a significantly lower ADR when compared to those not meeting these criteria (20.7% vs 48.2%, OR 0.30; 95% CI: 0.12–0.77). There was some variability in the ADRs of the individual attendings (range: 39.8% to 56.5%) however this was not found to be a significant predictor of overall ADR on multivariate analysis. Additionally, the size of the largest polyp detected in patients who failed CS by the strict definition was significantly larger (by 4.25 mm; p = 0.04) compared to those who did not fail CS. There was a non-significant decrease in the number of polyps resected per colonoscopy (of 0.73 polyps; p = 0.083). For patients who only met the expanded definition based on exceeding specified doses of fentanyl and/or midazolam, there was no difference between the groups in terms of ADR (42.8% for failed, 48.2% for not failed, p = 0.74), total number of polyps (p = 0.92), or the size of the largest polyp (p = 0.09).

## Discussion

This study reports several novel findings regarding the use of conscious sedation (CS) for patients undergoing an outpatient colonoscopy at an academic medical centre. We used a strict definition of CS failure in which the patient tolerated the procedure less than well or received a recommendation that future colonoscopies should be performed with monitored anaesthesia care (MAC) because of patient tolerability. Using this strict definition, we found a very low rate (3.8%) of CS failure. The rate of failed CS was still fairly low (22.8%) when using an expanded definition. We found that fellow involvement was associated with an increased likelihood of failed CS using both definitions. Interestingly, use of adjunct medications for sedation (e.g., diphenhydramine) was associated with both definitions for failed conscious sedation independent of associated risk factors (including age, outpatient use of opiate and/or benzodiazepine medications, history of alcohol abuse, or history of illicit drug use). Our study is also the first to report on the impact of failed CS on the adenoma detection rate as we found the ADR was substantially reduced in patients who met the strict definition of failed CS.

Our results for the impact of gender and fellow involvement are consistent with the relatively limited literature regarding conscious sedation for colonoscopy. Previous studies, including one from our institution, have previously shown women require higher doses of sedative medications for both upper and lower endoscopy and are more likely to report abdominal discomfort during colonoscopy^10,14–16^. The study by Czwornog also showed longer procedure time for female patients, which could also explain the need for higher doses of sedative medications. It has also been shown that, similar to our findings, procedures involving trainees are associated with higher doses of medications and produce higher pain scores^10,17^. There are many plausible explanations for this. Colonoscopies involving trainees can be up to 50% longer in duration, again possibly necessitating higher doses of medication based on total procedure time alone^18^. In addition, by virtue of their novice status, trainees likely lack the technical expertise to navigate turns in a comfortable manner, identify and reduce loops in the colon, and effectively and reliably perform other manoeuvres that minimize patient discomfort during colonoscopy.

Another novel finding in this study is the high predictive value for failed CS associated with the use of adjunctive medications for sedation (almost exclusively diphenhydramine). Unlike other predictors of failed CS at our institution, the decision to use an adjunct medication for sedation is universally determined after the endoscopist meets the patient immediately prior to the procedure. Although this would not be able to be incorporated into a pre-procedure tool, its value in this study lies more in what it signifies. Many of the factors that lead an endoscopist to decide to use diphenhydramine or other adjunctive medications are the very same variables found to be significant predictors of failure in this study (i.e. younger age, outpatient opiate use). Also notable is a brief report from Canada suggesting that diphenhydramine can cause paradoxical agitation in chronic cannabis users. It is possible that in this small group of subjects, the diphenhydramine itself is causing patients to fail CS^19^. The strong residual association between adjunctive medication use and failure of conscious sedation after adjusting for these confounders (including illicit substance use) indicates that in their pre-procedure assessment the endoscopist is accurately incorporating additional risk factors for individuals likely to fail CS. Further investigation is needed to help identify these additional risk factors, which could further improve a standardized risk stratification tool for which patients are likely to fail CS and would benefit from MAC.

The final novel association identified in this study relates to the adenoma detection rate (ADR). Likely because of its strong correlation with risk for interval colorectal cancer, the ADR has emerged as the most important quality measure in colonoscopy^20–22^. ADR has previously been linked to patient factors such as age, race, gender, comorbidities, and procedural factors – most notably withdrawal time and even endoscopist personality^23–26^. In addition, studies have also analysed differences in the ADR between sedated and unsedated patients with some showing clear benefit for sedation^27^ and others showing no difference^28,29^. Studies comparing ADRs in patients sedated with CS and MAC have demonstrated either no difference^30^ or a benefit to CS over MAC^29^. However, to our knowledge, ours is the first study to focus on the impact that poor tolerance of CS has on the ADR. Using the strictest definition of failure of sedation, we found significant differences in adenoma detection. Specifically, the ADR was 70% lower in patients who failed sedation by the strict criteria. To put this in perspective, the ADR went from a value that was almost twice the minimum acceptable ADR (25% as recommended by the ASGE) to a percentage that falls below this minimally acceptable ADR. It is worth noting that 2 patients who failed CS by the strict definition had procedures in which the cecum was not intubated. Although patients in whom the cecum was not reached would be expected to have a lower ADR, we found that even after excluding procedures in which the cecum was not reached, that patients meeting the strict definition for failed CS still had a significantly lower ADR. The size of the largest polyp in patients who failed sedation was more than 4 mm larger than in those who did not fail CS. Finally, there was a non-significant decrease in the number of polyps seen per colonoscopy for those who met the strict definition for failed CS. Taken together, these results suggest that small polyps are more likely to be missed in those patients who are inadequately sedated. These data make intuitive sense; as patients become more uncomfortable and/or unstable, attention is drawn away from performing a high-quality exam to tending to the patient. As a result, smaller polyps can get missed.

There are several possibilities about how to use this data moving forward. Given the performance of our predictive scores, these could be incorporated into a triage algorithm tasked with scheduling patients for CS or MAC. While it is not feasible to schedule all female colonoscopy patients with MAC, it may be worth exploring having fellows, especially early in their training, perform colonoscopies only on MAC patients. This would allow them to focus on the skills of colonoscopy itself without attention being distracted by sedation-related issues. Given the increased speed of MAC sedation, this may also increase throughput somewhat for endoscopy blocks that fellows participate in compared to those in which conscious sedation is used.

Our study has several strengths. We focused on the outpatient colonoscopy, which is the most common clinical scenario for a gastroenterologist. This eliminates many potential confounding variables involved with inpatient procedures (quality of bowel preparation, use of intravenous analgesic medicines, acute illness etc.) In addition, our use of a “strict” definition appears to be unique, as prior similar studies have used a composite definition like our expanded definition^10,31^. This strict definition solely focuses on patient tolerance of the procedure as determined by the endoscopist and is a very clean definition of failure of CS as high doses of medication alone do not necessarily indicate an unsuccessful sedation experience. The fact that only those meeting strict criteria for sedation had significant differences in the ADR metric further suggests that this is a clinically meaningful definition. Finally, this appears to be the first study to associate inadequate sedation and its negative impact on the ADR.

There are a few limitations to the study. As a retrospective cohort study, certain clinical parameters, especially substance abuse, may have been under-reported. In addition, medications were recorded from a medication list in the EMR and therefore, it is uncertain if patients were actually taking these medications. Another limitation is the subjective nature of determining patient tolerance. There are no set definitions for this and it is based solely on the endoscopist’s impression of how the patient did. It has been shown that endoscopists overrate how well patients tolerate procedures^32^. This, however, would then lead to an under-recognition of patients who would meet “strict” definition of CS failure. Furthermore, our strict definition may include patients, especially those in the “tolerated fairly well” category, who may have tolerated the procedure fine. We feel that by including in the strict definition all patients who tolerated the procedure less than well, we are eliminating as much subjectivity and heterogeneity from the definition as possible. In other words, despite differences in how individual endoscopists rate each category of tolerance, we are confident that all the patients excluded from the strict definition tolerated their procedures. Also worth noting is the somewhat arbitrary definition used for the expanded CS failure definition. These numbers are based on the amount of medication routinely available at the start of a colonoscopy at our centre. Receiving a dose greater than 5 mg of midazolam and/or 200 mcg of fentanyl does not necessarily indicate a true failure of conscious sedation. It is worth noting that the cut-offs used in this study are close to the maximum doses (6 mg of midazolam and 200 mcg of fentanyl) stated in the American Gastroenterological Association’s recent Review of Endoscopic Sedation^9^. Exceeding the “allowable” dose of medications can arguably be considered a failure of CS. Our study used an expanded definition of adenoma detection rate that included traditional tubular adenomas and sessile serrated adenomas/polyps. This was done to analyse the impact that failure of conscious sedation had on detection of clinically meaningful polyps regardless of the reason the procedure was performed. Indication of the procedure (diagnostic vs. screening/surveillance) was included in the logistic regression models for ADR. Finally, there are likely several variables that could potentially affect sedation tolerance and polyp metrics that were not measured in this study. Among those affecting the former are expertise of the endoscopist, decision to use a paediatric or an adult colonoscope and use of air or water insufflation and for the latter is inaccuracy in estimating polyp size from provider to provider. While these certainly could impact our outcomes, these variables are closely linked to the preference and practice habits of the performing endoscopist. By controlling for the individual endoscopists, we feel we have reasonably accounted for these variables. Another variable not captured in this study, quality of the bowel preparation, could have an impact on the ADR. However, its effect on sedation tolerance is unclear and since it is not something that can typically be predicted before starting a procedure, we did not include it in our analysis.

In conclusion, we demonstrated that failure of conscious sedation based on reported patient tolerability is uncommon, occurring in less than 1 in 4 colonoscopies performed at our institution during the study period using the most expansive definition. We identified female gender, trainee involvement, younger age, and the need for adjunctive medications as significant predictors. Future efforts to identify the additional patient factors that could be identified before the day of the procedure that lead the endoscopist to administer an adjunct medication for sedation would optimize the ability to triage patients who would benefit from going directly to MAC and those that are highly likely to do well with conscious sedation. Finally, the finding of a substantially reduced ADR in those who tolerate the procedure less than well deserves further investigation and consideration in determining the subsequent colonoscopy interval, similar to the current practice for patients with inadequate bowel preparation.
